# Dr. Edw. Register’s New Book

**Published:** 1907-05

**Authors:** 


### DR. EDW. REGISTER'S NEW BOOK.

"Practical Fever Nursing" will soon be issued from the presses of the W. B. Saunders Company, of Philadelphia. Dr. Register is well-known as the editor of the Charlotte Medical Journal, and as Professor of the Practice of Medicine in the North Carolina Medical College, at Charlotte, N. C. Dr. Register is a widely traveled, well-read, and a polished, dignified gentleman. He has always enjoyed a large and lucrative practice in his home city, as well as in nearby towns and in adjoining States. From his varied and ripe experience in the profession, the Doctor is in a position to write authoratively on the subject of "Fever Nursing."—Gaillard's Southern Medicine.

A deep ulceration of the fauces or tonsils should not be diagnosed as specific without examining the blood to exclude acute lymphatic lukemia.—American Journal of Surgery.

